# Maternal survival costs in an asocial mammal

**DOI:** 10.1002/ece3.8874

**Published:** 2022-05-11

**Authors:** Rachel Kanaziz, Kathryn P. Huyvaert, Caitlin P. Wells, Dirk H. Van Vuren, Lise M. Aubry

**Affiliations:** ^1^ 3447 Department of Fish, Wildlife, & Conservation Biology Colorado State University Fort Collins Colorado USA; ^2^ 3447 Graduate Degree Program in Ecology Colorado State University Fort Collins Colorado USA; ^3^ Department of Veterinary Microbiology & Pathology Washington State University Pullman Washington USA; ^4^ 8789 Department of Wildlife, Fish, & Conservation Biology University of California Davis Davis California USA

**Keywords:** demography, density, ground squirrel, kin, life history, sociality

## Abstract

Maternal characteristics, social dynamics, and environmental factors can all influence reproduction and survival and shape trade‐offs that might arise between these components of fitness. Short‐lived mammals like the golden‐mantled ground squirrel (GMGS; *Callospermophilus lateralis*) tend to maximize effort toward current reproduction at the expense of survival but may be complicated by other aspects of the species’ life history and environment. Here, we use 25 years of data (1995–2020) collected from a population of GMGS at the Rocky Mountain Biological Research Laboratory in Gothic, Colorado, to test the effect of several maternal characteristics (e.g., age, experience, and timing of litter emergence), social context (e.g., litter sex ratio and kin density), and environmental context (e.g., date of bare ground and length of vegetative growing season) on survival of reproductive female GMGS using Cox proportional hazard models. Our results indicated that social dynamics (i.e., density) and environmental conditions (i.e., standardized first day of permanent snow cover and length of growing season) explained significant variation in annual maternal survival, while maternal characteristics did not. A higher density of related breeding females and the total number of females (both related and unrelated to the focal mother) were associated with an increase in the mortality hazard. A later standardized date of the first day of permanent snow cover and a shorter growing season both reduced the maternal mortality hazard. Together, our results suggest that factors extrinsic to the squirrels affect maternal survival and thus may also influence local population growth and dynamics in GMGS and other short‐lived, territorial mammal species.

## Abstract 2

We used 25 years of longitudinal data collected from golden‐mantled ground squirrel females to test the effects of individual characteristics, the social context, and the environment on female mortality using Cox proportional hazard models. Both female kin density and the environmental context explained significant variation in female mortality, while individual characteristics did not. Our results suggest that both kin density and climate metrics related to growing season phenology swamp individual characteristics in explaining variability in maternal mortality. This is an important finding in a rather short‐lived species where every additional active season survived has a lot of weight in improving lifetime reproduction.

## 1 INTRODUCTION

Variability in the allocation of resources toward current reproduction, future reproduction, or survival (i.e., “the principle of allocation” (Levins, 1968)) can result in a fitness trade‐off known as the “cost of reproduction” (Roff, 1992; Stearns, 1992). When resources are limiting, a change in current reproductive effort affects an organism's fitness by inversely impacting survival and/or future reproduction (Reznick, 1985; Williams, 1966). Short‐lived mammals tend to maximize effort toward current reproduction at the expense of survival, whereas their long‐lived counterparts may delay reproduction and instead invest in survival, given greater reproductive flexibility (i.e., “bet‐hedging”) (Hamel et al., 2010; Starrfelt & Kokko, 2012; Stearns & Rodrigues, 2020). Maternal characteristics, social dynamics, and environmental factors all can influence female reproductive output and survival as well as the trade‐offs that may exist among these fitness traits (Williams, 1957).

Maternal characteristics that drive resource allocation for reproduction include individual characteristics (e.g., age of first reproduction and reproductive experience) that influence both reproductive output (e.g., litter size, sex ratio, offspring size, and quality) and annual survival (Authier et al., 2017; Hamel et al., 2010; Toni et al., 2020). Moore et al. (2016) studied the trade‐off between age of first reproduction and fitness from inter‐ and intra‐ generational perspectives in a population of golden‐mantled ground squirrels (*Callospermophilus lateralis*) (hereinafter GMGS) and found that delaying the age of first reproduction resulted in relatively lower individual fitness, measured as (i) lifetime reproductive success and (ii) individual fitness calculated as the dominant eigenvalue of the population projection matrix. In the bank vole (*Myodes glareolus*), previous reproductive status influences the mother's offspring sex ratio (Rutkowska et al., 2011), and in Columbian ground squirrels (*Urocitellus columbianus*), more experienced mothers had higher reproductive success than their inexperienced counterparts (Broussard et al., 2008). Studies on yellow‐bellied marmots showed adult survival was highest for reproductive females and for individuals with prior reproductive experience (Paniw et al., 2020).

The degree of sociality plays another important role in mammal life histories. The evolution of sociality is driven by the costs and benefits of close interactions with conspecifics, with sociality occurring when the benefits outweigh the costs, and *vice versa* for asociality (Boone, 2017; Silk, 2007). Costs related to group‐living include more predator attraction (Ale & Brown, 2007), increased transmission of pathogens and parasites (Cote & Poulin, 1995; Lucatelli et al., 2021), increased intra‐specific competition (Baalen & Yamauchi, 2019; Stockley & Bro‐Jørgensen, 2011), and increased stress (Creel et al., 2013). The benefits of social interactions include greater reproductive output (Armitage, 1987) and higher survival probability (Ebensperger et al., 2012). However, costs and benefits of social interactions may depend on whether interacting individuals are kin or nonkin. Golden‐mantled ground squirrels, the focus of this study, are classified as asocial (Armitage, 1981; Michener, 1983). Females defend territories and occupy burrows by themselves, but they may exhibit some home range overlap with close kin (i.e., mothers or daughters) (Aliperti, 2020; Jesmer et al., 2011).

Density and kinship together play a prominent role in shaping reproductive output and survival in GMGS. For example, the density of female GMGS within a population can dictate the sex ratio of offspring, favoring more daughters—the philopatric sex—when fewer adult females are present in the population (Wells & Van Vuren, 2017). Furthermore, density‐dependent offspring sex bias, favoring the production of sons when density is high, is more pronounced when the number of kin within the local area (i.e., “locality”) is higher (Wells & Van Vuren, 2017). The presence of littermate sisters can also affect reproduction by delaying the age of first reproduction, but mother and other kin presence has no effect for GMGS (Wells & Van Vuren, 2018). Although kin presence can affect fitness (Viblanc et al., 2010) and reproductive output (Barra et al., 2021; Wells & Van Vuren, 2017) in ground squirrels, its explicit link to survival has been less explored. Kneip et al. (2011) studied the effects of total female population density and environmental factors on survival in the same GMGS study system as Wells and Van Vuren (2017) and found that survival was lower with higher squirrel densities in the year prior, and short‐term survival was reduced by summer rainfall and predation pressure.

As mostly income breeders (Broussard et al., 2005) reproduction and survival rates of ground‐dwelling, hibernating mammals are especially influenced by variation in resource availability. When comparing similar‐sized mammals, species that hibernate typically have higher annual survival than their non‐hibernating counterparts (Turbill et al., 2011), but extended winter conditions can be detrimental to hibernating mammals if phenological mismatch leads to reduced resource availability at the time of emergence (Van Vuren & Armitage, 1991). In extremely seasonal environments, hibernation is a survival strategy that has evolved to buffer individuals during periods when resources are commonly lacking (e.g., alpine winter months). Variability in snow cover and rainfall in marmots, for example, contributes to variation in survival, likely because such conditions affect resource availability (Armitage, 2013). Even though hibernation increases annual survival overall, variation in resource availability during the active season plays an important role in determining adult mortality (Turbill et al., 2011). In early spring, above‐average precipitation can increase vegetative growth (Sharpe & Van Horne, 1998); however, rainy weather may preclude ground squirrels from seeking the resources needed for survival (Falvo et al., 2019; Kneip et al., 2011), perhaps because being wet challenges squirrel thermoregulation. High summer temperatures after the reproductive period also reduce foraging activity, resulting in diminished resource acquisition (Vispo & Bakken, 1993) which could reduce the probability of over‐winter survival.

Collectively, maternal characteristics interact strongly with the broader social and environmental contexts experienced to shape fitness trade‐offs (Gerber et al., 2021; Rusu & Krackow, 2004; Ziv & Davidowitz, 2019). We studied a population of female GMGS, an asocial, short‐lived, hibernating mammal species that is responsive to significant variability in maternal characteristics, social dynamics, and environmental factors, and assessed which factors had the strongest influence on maternal survival. We were particularly interested in understanding how reproductive investment varies among mothers and how it shapes their survival chances from year‐to‐year, within the contexts of extreme seasonality and complex social interactions.

## 2 METHODS

### 2.1 Focal species

Golden‐mantled ground squirrels are distributed throughout the Rocky Mountains along an elevational gradient from 1220 to 3965 m above sea level (Kneip et al., 2011). Spring emergence from hibernation occurs between early April and late May, and they enter hibernation in the fall, usually between late August and October (Bartels & Thompson, 1993; Bronson, 1980). Polygynous breeding takes place in early spring (Wells & Van Vuren, 2017). Females give birth to a single litter of one to nine altricial pups per year. Golden‐mantled ground squirrels can live up to seven to nine years (Bronson, 1980; Kanaziz, unpublished), but overall life expectancy is less than two years (Hostetler et al., 2012).

### 2.2 Study site and data collection

The Rocky Mountain Biological Research Laboratory (RMBL) is located in the East River Valley of Gunnison, Colorado (38°58'N, 106°59'W). The 13‐ha study site is situated at an elevation of 2900 m above sea level. A complete census of the GMGS population at RMBL began in 1990, with detailed pedigrees constructed for all adult females beginning in 1995. We used data collected from 1995 to 2020 on 141 resident females for which we had 249 annual observations and 131 mortality events. Females entered the study the year of their first reproduction, which occurred at age one or two for nearly all individuals. Because we only included reproductive females, any instance where an individual disappeared from the study site was classified as a mortality event as post‐breeding dispersal is extremely rare (<1% of females, Van Vuren, unpublished).

To develop a census database for analysis, field methods involved monthly trapping events during the active season and daily observations. Once trapped, individuals were identified by ear tag (Monel 1005‐1) and by unique dye mark (Nyanzol‐D) applied to the fur, weighed (g) with a Pesola scale, and assessed for reproductive status based on nipple development (Wells & Van Vuren, 2017). Individuals were classified as “alive” if they were trapped or seen during daily observations, but adult females who failed to return to the system were classified as “dead.” For immigrant individuals that were not born in the study site but entered and established a territory later, we used timing of entrance and mass at capture as indicators of age. Immigrants who were captured late in summer and had a mass consistent with other young of the year were classified as juveniles and assigned a known age; by contrast, immigrants who were captured in spring could be yearlings or older and mass could not be used as a categorical difference, making their age unknown.

### 2.3 Variables of interest and hypothesized relationships with survival

#### 2.3.1 Maternal characteristics

The census dataset and corresponding measurements were used to compile a list of variables related to maternal characteristics, including individual attributes as well as metrics of reproductive investment. Data were recorded in each year of the study for all female GMGS who were alive in that year. The ***age*** of each female was counted as the number of years since birth (i.e., a newborn juvenile was viewed as age 0; a yearling was age 1, etc.) with the expectation that increasing age would improve survival as ***experience*** (the difference between maternal age and age of first reproduction) increases, at least up to a point when senescence may lead to a decline in survival at older ages (e.g., Berger et al., 2016). ***Natal philopatry*** was determined based on whether a juvenile or adult immigrated into the study site, was born and bred in the same locality, or was born in the study site but moved from her natal locality to breed elsewhere within the study site. We expect resident mothers to experience better survival because site familiarity should give them an advantage in resource acquisition and predator avoidance as compared to their immigrant counterparts who came into the study site from a different population (typically as a juvenile or yearling). The ***age of first reproduction*** was recorded for all residents and, if an immigrant joined the system as a known juvenile, then her age of first reproduction was also recorded. By delaying age of first reproduction, a female can devote more resources to individual growth rather than invest in reproductive output, which should benefit her survival probability (Moore et al., 2016); 113 females started breeding at age 1 and 60 at age 2 or older in our dataset. ***Litter size*** was a count of the number of offspring that emerged from the natal burrow. We hypothesize that a smaller litter size (i.e., fewer offspring per litter) should be associated with higher maternal survival as maternal care is apportioned among fewer offspring. The ***litter mass*** was the cumulative mass of all offspring within a litter at emergence from the natal burrow (i.e., at weaning), and higher litter mass should be associated with lower annual survival, as allocation of energy to offspring growth may reduce female pre‐hibernation condition. The ***date of litter emergence***, a proxy of maternal reproductive timing, represents the difference (in days) between the date of litter emergence and the last day of spring permanent snow cover. Earlier litter emergence should improve maternal survival as mothers will have longer to prepare for hibernation after the reproductive period.

#### 2.3.2 Social context

The social environment is shaped by both reproductive output that can alter future social context as well as current density‐related factors that are especially important in asocial species like the GMGS. The number of daughters in each litter was used to calculate ***sex ratios***. Producing more daughters per litter (i.e., a female‐biased sex ratio) should be associated with lower survival probability as it increases the opportunity for daughter philopatry, which could increase kin overlap and intensify intraspecific competition. Female population size was considered on a local scale as the number of reproductive and non‐reproductive individuals within one of six discrete “localities” within the study site (Wells & Van Vuren, 2017). Higher local density of both kin and nonkin within the locality is hypothesized to result in lower maternal survival probability. Of particular interest was the breeding population as mothers are responsible for acquiring enough resources to sustain themselves as well as their offspring. Thus, the ***number of breeding females*** within the locality each year was also assessed (Wells & Van Vuren, 2017) with the logic that a higher density of breeding females will negatively affect maternal survival due to increased competition. Although interactions between related individuals tend to be less agonistic, females incur more competition for resources when they overlap home ranges with related kin (Aliperti, 2020). Such overlap leads to more intense intraspecific competition, which will likely translate to lower maternal survival probability, especially as the density of related females increases. Increasing the density of ***unrelated females*** should also be related to lower survival because interactions against nonkin are more aggressive, which negatively affects survival as shown in other asocial mammals (Cubaynes et al., 2014). Overall, higher ***local population density*** will likely result in lower survival probability because of increased intraspecific competition for resources and aggression.

#### 2.3.3 Environmental context

Weather variables pertaining to snow data were obtained courtesy of Billy Barr (2020), and variables for rainfall and temperature were derived from the National Weather Service Forecast Office for Crested Butte, Colorado (NOAA, 2020; https://www.noaa.gov/tools‐and‐resources/weather‐andclimate‐resources). The ***first day of bare ground*** marked the onset of vegetative growth, signifying the beginning of food availability for GMGS. However, early snowmelt is also associated with drought and reduced primary productivity (Sloat et al., 2015). To determine the end point of the active season, the first ***day of permanent snow cover*** served as the initial day of snow after which the ground stayed continuously covered; this day was also standardized by the number of days past April 30. An intermediate first day of permanent snow cover will have the greatest positive effect on squirrel survival as too early snow cover might not give squirrels time to acquire enough resources to survive over‐winter, but too late snow cover may reduce thermal cover, leaving burrows less insulated. The duration of days between the date of bare ground in the spring and the last snow‐free day before permanent cover in the fall for each year represented the ***length of the vegetative growing season*** (Schwartz & Armitage, 2005). An intermediate length of the growing season should optimize survival as individuals need enough time to acquire the resources needed for over‐wintering but not so long that drought conditions prevail and reduce vegetative quality.

Snowfall was measured with a ruler to record depth (cm) of the snow from a snowboard adjacent to the study area. The snowboard was cleared each morning and afternoon after measurement such that daily snowfall was the sum of the morning and afternoon measurements (Barr 2020). ***Total winter snowfall*** was calculated by adding monthly totals from September to July beginning the fall prior to the year assessed (e.g., total snowfall associated with the 1995 active season was calculated based on the snowfall from September 1994 through July 1995). Too little snowfall reduces snowpack and increases the risk of freezing to death (Cordes et al., 2020), but too much snowfall can lengthen the duration of hibernation which could be fatal if squirrels do not acquire enough body fat to endure an extended hibernation (Armitage, 2013); thus, an intermediate amount of snowfall is expected to optimize survival.

During the months of June and July each year, the ***total amount of summer rainfall*** was recorded in centimeters (Kneip et al., 2011). Rainfall is necessary for vegetative growth, but it reduces the amount of time squirrels spend above‐ground acquiring resources and can cause burrow flooding. In addition, low rainfall totals can lead to drought‐induced food limitations which, in turn, reduce the chances of over‐winter survival for hibernating squirrels (Farand et al., 2002). To balance these aspects, survival is expected to be highest when there is an intermediate amount of rainfall. Summer temperature can also affect the length of time squirrels spend actively acquiring resources. The ***average summer temperature*** during June and July was calculated in degrees Celsius. Lower summer temperatures should improve survival as higher temperatures reduce foraging time and increase energy expenditure as squirrels must avoid overheating (Cordes et al., 2020; Fletcher et al., 2012). In addition, higher summer temperature can cause vegetative quality to decline by causing drought (Armitage, 2013). Further, the ***number of summer days with temperatures above 25 degrees Celsius*** were counted during June and July (if data were missing, then it was assumed the temperature was less than 25°C) as temperatures higher than that reduce above‐ground activity in at least one other species of ground‐dwelling squirrels (i.e., alpine marmots, *Marmota marmota*) (Turk & Arnold, 1984) and may do so for GMGS, too. Both of these activities reduce the time available for acquiring resources which we expect will have a negative effect on survival.

### 2.4 Data analysis

Because all females in the study site are detected and observed every year, from first occurrence in the study site until death, the assumption of perfect detection is met for the life of each tracked individual; thus, one can use known‐fate survival models for estimating annual survival rates (e.g., Wintrebert et al. 2005). We used known‐fate Cox proportional hazard models (CPH; Cox, 1972), an extension of the non‐parametric Kaplan–Meier model (1958), to estimate annual survival as a function of the covariates of interest discussed above (e.g., Aubry et al., 2011). Cox proportional hazard models are semi‐parametric and allow the mortality hazard to fluctuate with time while measuring the effects of covariates on either age‐ or time‐specific survival (Klein, 1992). Cox proportional hazard models make no assumption about the shape of the underlying mortality hazard (i.e., the “force” of mortality) over time. Each covariate within the model is assumed to act multiplicatively (i.e., proportionally) on the baseline mortality hazard at each time step (e.g., Bradburn et al., 2003), such that:

$$h_{0}\left(t\right)\;\times \;h\left(t,{\;X}_{i}\right)=\exp\left(\sum_{i=1}^{p}\beta_{i}X_{i}\right),$$

where *h*_0_ refers to the baseline hazard (i.e., the hazard's value when all covariate values are null), *p* denotes the number of parameters in the model, the *β*s denotes a set of estimated parameters, and the *X*s represents the data, or series of covariate values for each individual *i* such as *X* = (*X*_1_, *X*_2_,…*X*_i_), and *t* denotes time (in this case, time elapsed since first reproduction rather than actual age). *X_i_* can either consist of one unique value per individual (e.g., the age at first reproduction) or can be a vector of values (i.e., one value per year lived for each individual as, for example, with time‐specific reproductive investment).

As mentioned above, Cox proportional hazard models allow the mortality hazard to fluctuate with time while measuring the effects of covariates on time‐specific survival. In our analysis, we tested for the effect of covariates of interest measured at time *t*, where *t* encompasses the active season in a given year (e.g., kin density in active season 2010) on survival from *t* to *t* + 1 (e.g., the focal mother survived from spring emergence 2010 until spring emergence 2011). Because a fair portion of this interval overlaps with hibernation, mortality could only be assessed if an animal did not emerge from hibernation the following spring (*t*+1).

An individual's contribution to the mortality hazard consists of an entry time, an exit time, and whether the animal survived the interval. We measured the effect of any given covariate at time *t* on the likelihood of an individual surviving the interval from *t* to *t*+1 (i.e., from spring of a given year to the following spring), as opposed to surviving the animal's entire lifespan, which would range across multiple years in some cases. All analyses were performed in RStudio (R Core Team, 2019), using the “survival” package (Therneau & Grambsch, 2000) as well as the “survminer” (Kassambara & Kassambara, 2020) and “AICcmodavg” (Mazerolle & Mazerolle, 2017) libraries. The function “cox.zph” was used to assess the proportional hazard assumptions of the model to ensure covariates weighed evenly on the mortality hazard (e.g., Aubry et al., 2011).

Finally, although we worked with an extensive dataset in terms of its longitude, we had to contend with rather small sample sizes year‐to‐year (see Appendix SA for details) such that a classic backward and stepwise model selection process was not possible; the most complex models that encompassed all effects of interest did not converge on model parameters and provided unreliable estimates. Here, we report the results of less complex yet supported models exploring the effects of variables related to maternal characteristics, social context, and the environment on golden‐mantled ground squirrel survival.

## 3 RESULTS

### 3.1 Maternal characteristics

We tested for the effect of different parameterizations of age (i.e., a continuous parameterization was compared to 2 age classes “1, 2+”, 3 age classes “1, 2, 3+”, and 4 age classes “1, 2, 3, 4+”), experience (i.e., a continuous parameterization was compared with 2 age classes “0, 1+”), natal philopatry status (i.e., resident versus immigrant), age at first reproduction (i.e., a continuous parameterization was compared with 2 age classes “1, 2+”), litter size (i.e., a continuous parameterization was compared with the following categories “0, 1+”, “0, 1–3, 4–5, 6+”), litter mass (i.e., a continuous parameterization was compared with a categorical one “lite, moderate, and heavy”), and day of litter emergence (i.e., a continuous parameterization was compared with a categorical one “early, moderate, and late”) on maternal survival, because we had no *a priori* reason to support a continuous fit over a categorical one. None of the best performing models evaluated for maternal characteristics (Appendix SB1) suggested a significant relationship between the variables of interest and maternal survival.

### 3.2 Social context

Using different parametrizations of sex ratio (i.e., the standardized litter size sex ratio of female to male offspring, where positive values indicate more daughters and negative values represent more sons), nonbreeding female density (i.e., continuous; 0–1, 2+; 0, 1–2, 3+), unrelated, related, and total breeding female density (i.e., continuous; 0–1, 2+; 0, 1–2, 3+), we tested for an effect on the maternal mortality hazard. We did not have any *a priori* knowledge to suspect that a linear relationship between density‐related covariates and maternal mortality would outperform a categorical effect of density‐related covariates on maternal mortality; therefore, we compared these different parameterizations to determine which would best fit the data based on model selection.

**FIGURE 1:**
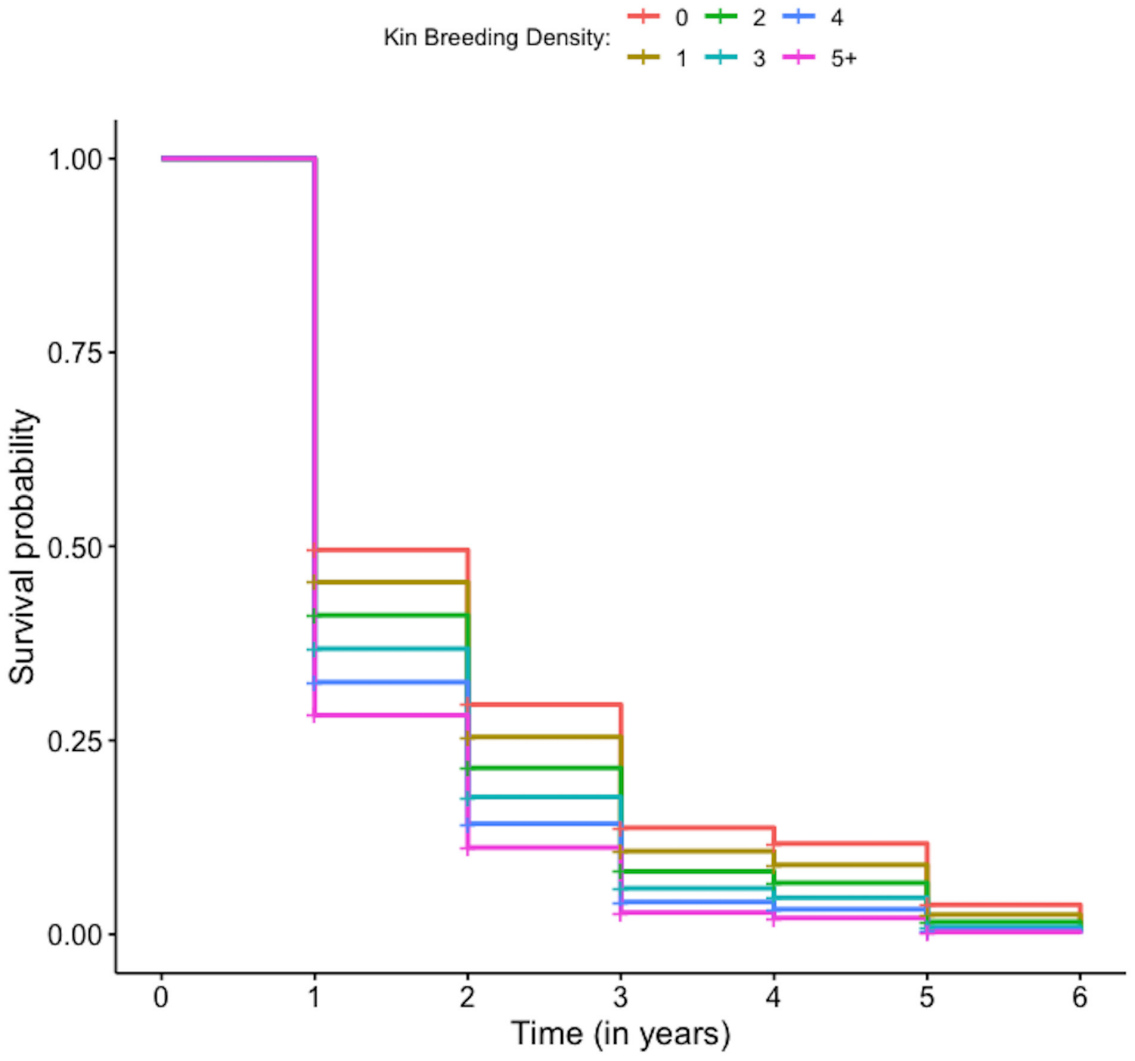
Effect of the number of breeding kin density (i.e., 0, 1, 2, 3, 4, 5+) within one of six discrete “localities” assigned to all females (Wells & Van Vuren, 2017; see Methods for details) on the annual survival of a golden‐mantled ground squirrel population studied at the Rocky Mountain Biological Station, in Gothic, Colorado, from 1995 to 2020. 95% confidence intervals are not presented here for the sake of clarity (too many kin breeding density groups are presented)

Among the top models (Appendix SB2), we found that related (kin) breeding female density and total female density had a significant effect on maternal survival (Table 1, Figure 1). Looking at the density of related breeding females, kin presence treated as a continuous variable ranked better than other structures of kin presence ((0–1, 2+) or (0, 1–2, 3+)) in describing variability in the maternal mortality hazard (Appendix SB2). Kin (continuous) included a range of 0–5 individuals, and marginally significant results indicated that living near breeding kin positively affected mortality, as every additional related female led to a 12.5% increase in the mortality hazard (HR = 1.125; 95% CI = [0.9812, 1.289]; *p*‐value = .091; Table 1, Figure 1). We found similar results for total local female density (both related and unrelated) in that the population size (continuous) model outcompeted the discrete population size models ((0–1, 2+) and (0, 1–2, 3+)) for representing variation in maternal survival (Appendix SB2). Total local female density ranged from 0 to 11 individuals, and we found marginally significant support that increasing the local total female density by one unit increased the mortality hazard by 6.3% (HR = 1.063; 95% CI = [0.993, 1.138], 1.14; *p*‐value = .079; Tables 1, 2viii).

**TABLE 1:** Hazard ratios and 95% confidence intervals obtained from the best performing Cox proportional hazard models testing for the effect of social context, specifically litter size (i), litter sex ratio (ii), collective litter mass (iii), day of litter emergence (iv), local female density (v), density of related (kin) breeding female (vi), local density of unrelated (nonkin) breeding females (vii), and local population density of total breeding and nonbreeding females (viii)

	Selected Models (see Appendix SB3)	Hazard Ratio	95% Confidence Interval	*p*‐value^a^
(i)	Litter size (Continuous)	0.967	0.876, 1.074	.556
(ii)	Ratio (Continuous)	0.863	0.692, 1.076	.19
(iii)	Mass (Continuous)	0.999	0.996, 1.003	.826
(iv)	Emergence (Continuous)	1	0.981, 1.020	.996
(v)	Breeders (0–1, 2+)	1		
	Breeders (Continuous)	1.28	0.887, 1.848	.187
(vi)	Kin (Continuous)	1.125	0.981, 1.289	.091
(vii)	Nonkin (Continuous)	0.998	0.876, 1.116	.851
(viii)	Population (Continuous)	1.063	0.993, 1.138	.079

^a^ The p‐value is from a test on the null hypothesis of no difference in the risk of mortality between the reference variable and a given variable; if *p*‐value < .05, the null hypothesis is rejected and a difference between variables is deemed statistically significant, marginally significant if <0.1

^b^ Results were evaluated on a continuous scale ranging from 99 to 615 g.

**TABLE 2:** Hazard ratios and 95% confidence intervals obtained from the best performing Cox proportional hazard models testing for the effect of environmental context, specifically the standardized date of the first day of bare ground (i), the standardized date of the first day of permanent snow cover (ii), the length of the growing season (iii), the amount of winter snowfall (iv), the amount of summer rainfall during June and July (v), the average summer temperature during June and July (vi), and the number of days above 25°C during June and July (vii)

	Selected Models (see Appendix SB3)	Hazard Ratio	95% Confidence Interval	*p*‐value^a^
(i)	Bare ground—early (Reference)	1	‐	‐
	Bare ground—moderate	1.14	0.667, 1.957	.628
	Bare ground—late	1.47	1.013, 2.142	.042
(ii)	First day of perm. snow cover	0.983	0.966–0.999	.045
(iii)	Season length—short (Reference)	1	‐	‐
	Season length—medium	1.433	0.972–2.114	.0692
	Season length—long	0.886	0.528, 1.486	.646
(iv)	Snowfall—low (Reference)	1	‐	‐
	Snowfall—moderate	0.942	0.628, 1.413	.773
	Snowfall—high	1.299	0.825, 2.044	.259
(v)	Rain—Continuous	0.890	0.741, 1.07	.210
(vi)	Temperature—Continuous	1.1492	0.948, 1.392	.156
(vii)	25°C days—Continuous	1.015	0.992, 1.030	.269

^a^ The p‐value is from a test on the null hypothesis assuming that there is no difference in the mortality hazard between the reference level and any other level considered; if *p*‐value < .05, the difference is deemed statistically significant, marginally significant if <0.1

### 3.3 Environmental context

We evaluated different parametrizations (i.e., continuous; early, moderate, and late) of date of bare ground, first day of permanent snow cover, length of the vegetative growing season, amount of winter snowfall, amount of summer rainfall during June and July, average summer temperature during June and July, and number of days above 25°C on maternal survival.

**FIGURE 2:**
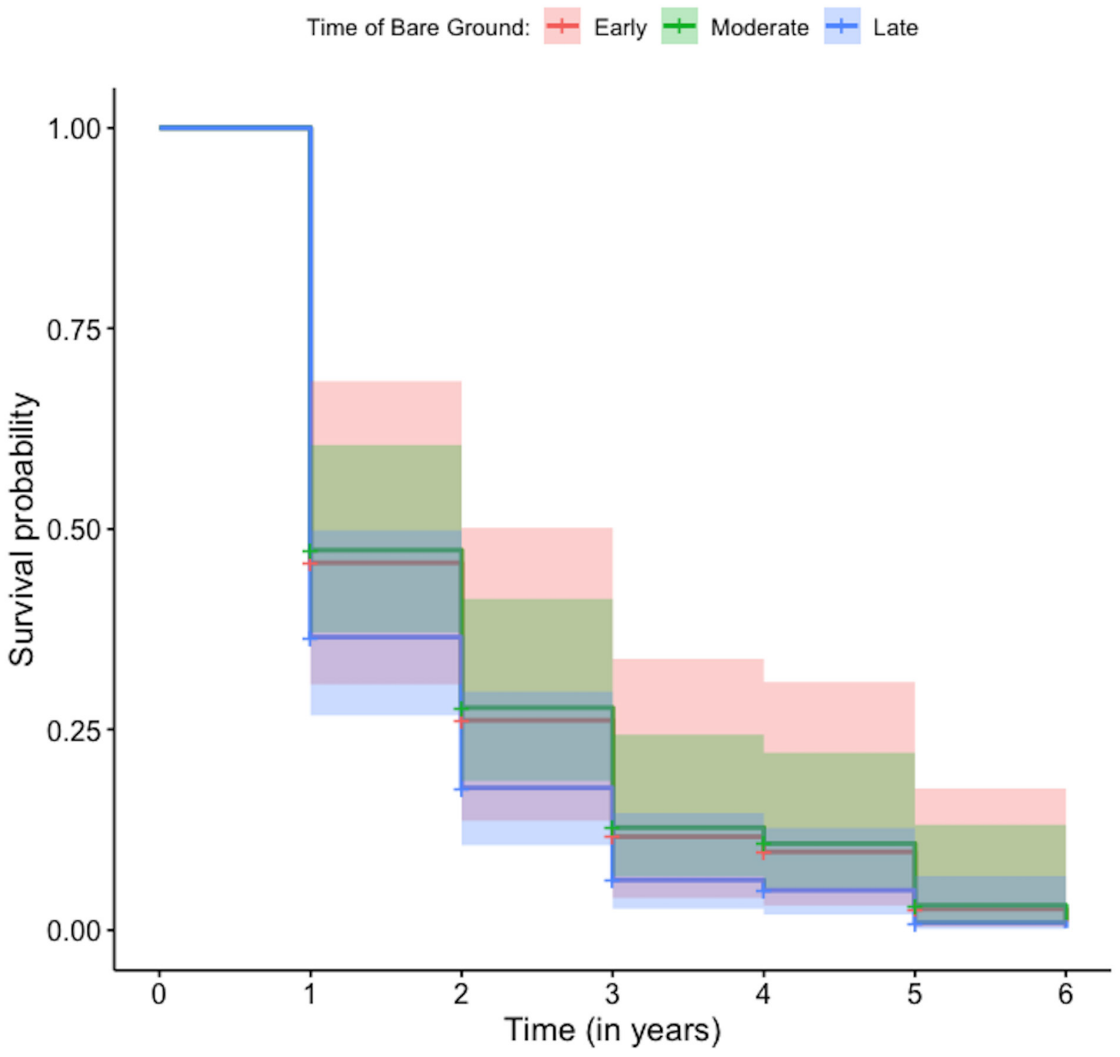
Effect of the timing of bare ground in spring (i.e., early, moderate, or late) on the annual survival of a golden‐mantled ground squirrel population studied at the Rocky Mountain Biological Station, in Gothic, Colorado, from 1995 to 2020. 95% confidence intervals are presented in shaded color

**FIGURE 3:**
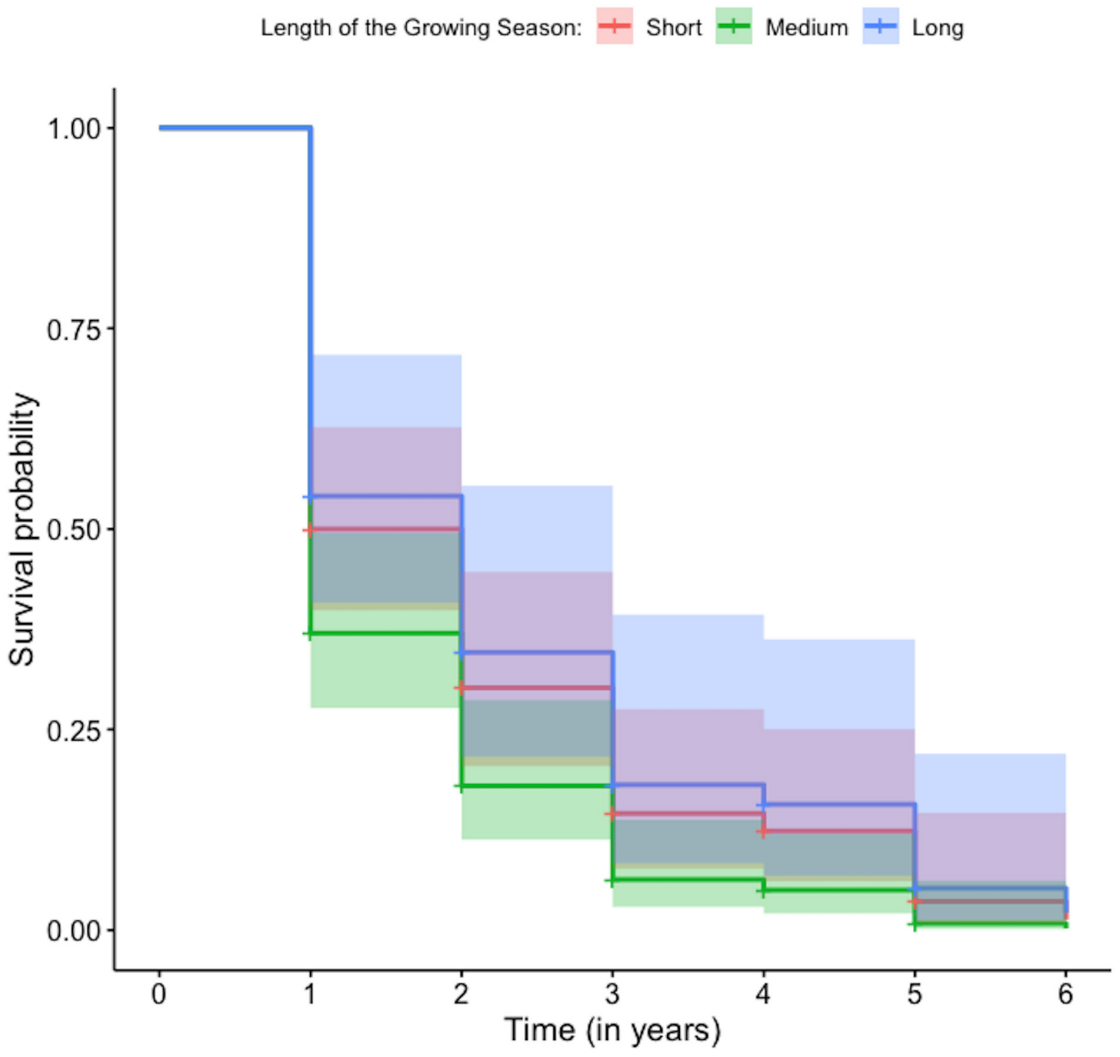
Effect of the length of the growing season (i.e., short, medium, or long) on the annual survival of a golden‐mantled ground squirrel population studied at the Rocky Mountain Biological Station, in Gothic, Colorado, from 1995 to 2020. 95% confidence intervals are presented in shaded color

The best performing models (Appendix SB3) that had a significant effect on the maternal mortality hazard included the date of bare ground, date of snow cover, and the length of the growing season (Table 2). The date bare ground in spring treated as a categorical variable (i.e., early, moderate, and late) ranked better than the continuous parameterization model in accounting for variation in the maternal mortality hazard (Appendix SB3). A late date of bare ground in spring (>May 25) increased the mortality hazard by 47% when compared to an early first date of bare ground (April 24–May 8) (HR = 1.47; 95% CI = [1.013, 2.142]; *p*‐value = .042; Table 2–ii; Figure 2). Snow cover treated as a continuous variable ranked better than the categorical parameterization (i.e., early, moderate, and late) model in accounting for variation in maternal survival (Appendix SB3). The first date of permanent snow cover ranged from October 17 to November 21 in a given year, yet GMGS enter hibernation as early as late August, especially at higher elevations (Bartels & Thompson, 1993). Model results indicated that delays in the timing of permanent snow cover caused a 2% decline in the mortality hazard (HR = 0.983; 95% CI = [0.966, 0.999]; *p*‐value = .045; Table 2) for each additional day that the study site remained snow‐free. The categorical parameterization (i.e., short, medium, and long) of length of the growing season outperformed the continuous parameterization in explaining variability in maternal survival (Appendix SB3). There was marginal evidence that an average season length (range: 158–182 days) increased the mortality hazard by 43.35% when compared to a short season length (range: 135–158) (HR = 0.4335; 95% CI = [0.972, 2.114]; *p*‐value = .0692; Table 2–iii; Figure 3), although that results were only marginally significant. But, a longer than average growing season (range: 182–204) did not significantly affect the mortality hazard (Table 2–iii; Figure 3).

Interactive models tested whether local density could mediate the relationship between litter characteristics (i.e., litter size, emergence timing, and sex ratio) and maternal survival but none of the effects were significant (results not presented here for the sake of conciseness).

## 4 DISCUSSION

Our study sheds light on how the presence of kin may affect maternal survival, a key component of local population growth and dynamics in short‐lived territorial species where females tend to remain philopatric. Our results further suggest that changes to both the social context and climate could shift life history patterns in a relatively short‐lived species. Indeed, within this population of GMGS, variability in maternal survival is affected by both the social and environmental context experienced by mothers. Notably, the mortality hazard increased as the density of kin breeding females increased. Mothers also experienced a reduced mortality hazard when the first day of permanent snow cover during autumn was delayed, and increased mortality when either spring snowmelt was late or the length of the vegetative growing season was moderately long.

### 4.1 Maternal characteristics

None of the maternal characteristics we tested had an effect on maternal survival. As a short‐lived species, few GMGS individuals may reach the point at which senescence begins to affect survival, which could explain the lack of effect between age and annual survival (Turbill & Ruf, 2010). Likewise, breeding experience may not have affected GMGS annual survival because, although they are iteroparous, their short lifespan means that all mothers tend to attempt breeding each subsequent year after their first reproductive attempt. This differs from the reproductive strategies of long‐lived species who may not breed every year yet experience higher survival with increasing experience (Aubry et al., 2011). We only included individuals once they made their first attempt at breeding, thus any individuals that died before they had any reproductive experience were not included in our analysis, and their effect on annual survival was not considered. We did not detect a difference in maternal survival among females that had successfully weaned at least one litter (*n* = 212) versus those that failed to wean one (*n* = 7) most likely because few individuals failed to wean a litter once they started to breed. Despite not detecting an effect on maternal survival, females that skipped breeding or were unsuccessful in producing a litter were most likely in poor condition and unable to sustain the energetic investment required for reproduction (Festa‐Bianchet & King, 1991; Murie & Dobson, 1987). Although producing a litter with any number of offspring requires an additional investment of resources by mothers, the ability to wean a litter suggests that those mothers were in better condition and thus able to support reproductive effort without incurring a cost to survival (Festa‐Bianchet & King, 1991; Murie & Dobson, 1987). Our results support the growing body of work that maternal survival of ground squirrels may be more affected by body condition and environmental conditions than the cost of producing a litter (Festa‐Bianchet & King, 1991; Hare & Murie, 1992; Neuhaus, 2000; Risch et al., 2007; Rubach et al., 2016; Skibiel et al., 2013).

When studying the effect of resident status on annual survival, we expected that site familiarity would give residents a substantial advantage (e.g., more efficient resource acquisition and better predator avoidance) over immigrants (Clutton‐Brock & Lukas, 2012). A limitation in our study was that we were only able to compare residents and immigrants within the study site, but we were unable to track dispersers that left the study site and may have had a different mortality hazard during the dispersal period. It is expected that dispersal is a risky behavior with higher mortality during the act of dispersal (Byrom & Krebs, 1999; Garrett et al., 1988; Van Vuren & Armitage, 1994). As it stands in our study, the lack of difference in survival between residents and immigrants indicates that, once a mother is able to establish a territory within a population and have her first reproductive event, her survival probability from that point onward is the same regardless of her prior residency status.

We expected that survival would be higher when individuals delayed reaching reproductive maturity (Descamps et al., 2006), but we found no difference in annual survival. Our finding differs slightly from Moore et al. (2016) who found that delaying the age of first reproduction causes a significant decline in fitness, possibly through a decline in survival, for species with faster‐paced life histories, but claimed that such an effect may be counteracted by the benefits of early maturity. Our results indicated no effect of delayed reproduction on survival. Again, this could be in part due to the design of our study in which we only included females once they became reproductively active; individuals in poor body condition that delayed reproduction but did not survive to ever breed were not included in our analysis.

Litter mass represents the amount of resource investment that mothers put into their litters. Heavier litters indicate greater investment which we thought would weaken maternal condition and, therefore, reduce maternal survival. However, we found no effect of litter mass on annual survival, which suggests that mothers adjust their investment according to their individual body condition (i.e., a mother in poorer condition will invest less than a mother in better condition who can invest more) as evidenced by the positive correlation between increasing maternal body fat (i.e., condition) and increasing litter mass (Robbins et al., 2012). As has been demonstrated experimentally in Columbian ground squirrels (Hare & Murie, 1992), GMGS mothers may invest as much as they can into their litters without detracting from their own body condition, nor risking a decline in their own survival. We examined a subset of the females for whom we had body mass data at spring emergence and found no relationship between this metric of body condition and maternal survival (see Appendix SC); nevertheless, our results highlight the importance of further exploration of body condition, reproductive investment, and subsequent survival.

We expected that mothers would have higher survival when they had more time after the reproductive period to prepare for hibernation to improve their body condition by gaining fat deposits necessary for over‐winter survival (Dark, 2005). Thus, earlier timing of litter emergence was anticipated to improve maternal survival. Instead, we found no significant effect on annual maternal survival regardless of when litters emerged (Table 1, model iv, *p*‐value = 0.996). However, timing of emergence may have a more influential effect on offspring survival (Armitage et al., 1976; Rieger, 1996). Later emerging Uinta ground squirrel (*Spermophilus armatus*) offspring tend to emerge at a heavier weight (Rieger, 1996), but the rate of mass gain is significantly reduced for GMGS juveniles that emerge later in the summer (Wells & Van Vuren, 2018).

### 4.2 Social context

Litter sex ratio did not affect maternal survival despite the hypothesis that the production of more daughters would generate increased competition for resources. While raising more of the philopatric sex can indeed lead to more intense competition (Clark, 1978), GMGS mothers have male‐biased litters when female kin density is high (Wells & Van Vuren, 2017), which may help compensate for the anticipated effect of higher female density. By adjusting the sex ratio of their litters, mothers may preemptively avoid the opportunity that daughters will generate more competition, thereby explaining the lack of effect we found in our survival model.

It is not surprising that total density negatively affected maternal survival. As an asocial species, we expected that one of the costs GMGS would incur by interacting with conspecifics would be a reduction in survival, likely due to increased resource competition (Aliperti, 2020; Stockley & Bro‐Jørgensen, 2011). Breeding females are likely more sensitive to more intense competition for resources as they are primarily considered income breeders, relying on daily energy intake (Stearns, 1992) after parturition (Broussard et al., 2005; Wells & Van Vuren, 2017). The local density of related females also decreased maternal survival. In fact, this negative effect of the number of female kin was even greater than that of total female density on maternal survival. Because the number of nonkin female did not have a significant effect on maternal survival, the number of female kin may be driving the observed negative effect of total female density on maternal survival. Golden‐mantled ground squirrel females may establish and defend territories (Michener, 1983), but they do exhibit some degree of home range overlap with other females (Jesmer et al., 2011), especially relatives (Aliperti, 2020). Related females likely experience some fitness benefit by overlapping home ranges (e.g., inclusive fitness (Armitage, 1987)), but our results suggest that they experience a cost to their survival as kin density increases (i.e., marginal effect of the density of related (kin) breeding female on the maternal mortality hazard; Table 1, model vi), which may be partially explained by increased competition for resources within a shared home range. The extra energy required for territory defense may leave reproductive females, who are already investing heavily in reproduction, more susceptible to a greater risk of mortality.

These results emphasize the asocial aspect of the GMGS life history as we see that, although related females may tolerate each other within their home ranges, they do so at a cost to their own survival. Mother–daughter pairs show greater overlap than sisters (Aliperti, 2020), and it would be interesting to distinguish between the role mother–daughter versus sister bonds play in shaping maternal mortality costs in future work.

### 4.3 Environmental context

The standardized date of the first day of bare ground fluctuated within a 58‐day range, while the standardized date of the first day of permanent snow cover fluctuated within a 35‐day range, and both exhibited a significant effect on the maternal mortality hazard when delayed, but in opposite direction. A delay in the timing of spring bare ground significantly increased the maternal mortality hazard, while a delay in the timing of permanent snow cover in fall led to a slight decline in the maternal mortality hazard.

Hibernating ground squirrels can show flexible responses to environmental variation and may match their phenology to changing environmental conditions (Sheriff et al., 2011; Williams et al., 2014). Yet, our results suggest that female golden‐mantled ground squirrels may not always be able to adjust the timing of their emergence to match optimal environmental conditions, creating a mismatch between the timing of resource acquisition and vegetative growth, substantially increasing the maternal mortality hazard. Indeed, a late date of the first day of bare ground in spring (>May 25) increased the mortality hazard by 47% when compared to an early first day of bare ground (April 24–May 8).

Our results also support the idea that a shorter growing season can improve maternal survival as mortality risk is lower during the hibernation period (e.g., reduced predation and reduced cellular aging) (Bieber et al., 2014; Kirby et al., 2019; Turbill et al., 2011). Reproductive females in particular must meet the energetic demands of reproduction by increasing the amount of time they spend foraging (Macwhirter, 1991). Although reproductive females may incur a survival benefit from having a longer growing season during which they can acquire more resources to compensate for reproductive output and better prepare for hibernation, an average season length may lead to a higher maternal mortality hazard when compared to a shorter than average growing season. This result suggests that the mortality costs of being exposed to predators, and other sources of mortality exceed the benefit of a longer growing season for reproductive females to some extent (note that a longer than average growing season did not have a significant effect on the maternal mortality hazard). A recent study supports this hypothesis and found that among 82 different mammalian hibernating species, longevity increased significantly with hibernation season duration, an effect that was particularly strong in small hibernators (Constant et al., 2020). This confirms that hibernation not only allows small mammals to survive periods of energy scarcity, but further suggests a reduction in mortality risk for those species that spend more time belowground (Constant et al., 2020). An additional hypothesis is the idea that the quality of the vegetation in a long growing season decreases over time, and as plants (graminoids and forbs) grow, their lignin content increases, making it harder to process as well as less nutritious. This idea has been tested in other species and different ecosystems (e.g., Aubry et al., 2013), but remains to be looked at in our study.

None of the weather variables that we evaluated had an effect on annual survival. We looked at winter snowfall as a representation of snow cover (Armitage, 2013), but did not consider snowpack (Ikeda et al., 2021). We know that snow conditions can affect fitness in other ways: snowpack is an important condition necessary for maintaining appropriate hibernaculum temperature for over‐winter survival (Tafani et al., 2013) and is known to affect reproductive output (e.g., litter size (Tafani et al., 2013)), but we did not detect a relationship between snowfall and annual survival. In future, it may be beneficial to study the effect of snowpack rather than simply snowfall.

During the active season, moderate weather conditions for rainfall and temperature, particularly with fewer days above 25°C (Turk & Arnold, 1984), should produce favorable conditions that optimize survival for ground‐dwelling squirrels (Schwartz & Armitage, 2005). We did not detect an effect on survival for either rainfall or temperature within each year. This differed slightly from the results of Kneip et al. (2011) who found a significant negative effect of rainfall on juvenile survival and suggested a potential positive effect on adult survival. Given the results of both studies, it appears important to consider age and potentially sex in relation to rainfall and survival. Because our study included only reproductive females who have established territories, they may be more efficient at acquiring resources throughout the active season as opposed to juveniles who have fewer days to forage and gain mass necessary for over‐winter survival.

### 4.4 Conclusions

Collectively, our results provide further insight into the impacts of various maternal, social, and environmental components on maternal survival. Our results may enable us to predict future population‐level effects of changing conditions as we anticipate that continuing climate‐mediated environmental variability will affect maternal survival. It also begs the question of how these results might change depending on the age, sex, and/or status of other members within the population beyond adult, reproductive females. Our study enhances understanding of the spectrum of sociality by shedding light on how relatedness and associated interactions may affect maternal survival, a key component of local population growth and dynamics in short‐lived territorial species, where females tend to remain philopatric.

## CONFLICT OF INTEREST

The authors have no conflict of interest to declare.

## AUTHORS’ CONTRIBUTION

**Rachel Kanaziz:** Conceptualization (lead); Data curation (equal); Formal analysis (equal); Funding acquisition (lead); Investigation (lead); Methodology (equal); Project administration (equal); Writing – original draft (lead); Writing – review & editing (supporting). **Kathryn P. Huyvaert:** Conceptualization (equal); Investigation (equal); Methodology (equal); Project administration (equal); Writing – review & editing (equal). **Caitlin P. Wells:** Conceptualization (equal); Data curation (lead); Investigation (equal); Methodology (equal); Project administration (equal); Writing – review & editing (equal). **Dirk H. Van Vuren:** Conceptualization (supporting); Data curation (lead); Investigation (equal); Methodology (supporting); Project administration (supporting); Writing – review & editing (equal). **Lise M. Aubry:** Conceptualization (lead); Data curation (supporting); Formal analysis (lead); Funding acquisition (equal); Investigation (equal); Methodology (equal); Project administration (equal); Writing – review & editing (lead).

## Supporting information

Supplementary Material

Supplementary Material

Supplementary Material

## Data Availability

The mortality hazard estimates and associated standard errors are reported in the main text and in the supplemental information. The data, covariates, and R code have been archived in Dryad (https://doi.org/10.5061/dryad.crjdfn36g).
