# Supplementary material for: Maternal survival costs in an asocial mammal
Source: Ecol Evol. 2022 May 11;12(5):e8874. doi: 10.1002/ece3.8874 (PMC9092287; doi:10.1002/ece3.8874)

# **MATERNAL SURVIVAL COSTS IN AN ASOCIAL MAMMAL**

**Appendix A. Data description.**

We used data collected from 1995 to 2020 on 141 resident female golden-mantled ground squirrels studied at the Rocky Mountain Biological Laboratory (RMBL) in Gothic, Colorado, for which we had 249 annual observations and 131 mortality events. Table A1 provides the breakdown of sample size over the span of the study and represents the number of females that were alive, or reported dead, in any given year.

**Table A1.** Sample size by year, providing the number of females reported alive versus dead each year at the Rocky Mountain Biological Station, in Gothic, Colorado, from 1995 to 2020.

|  | 1995 | 1996 | 1997 | 1998 | 1999 | 2000 | 2001 | 2002 | 2003 | 2004 | 2005 | 2006 | 2007 |
| --- | --- | --- | --- | --- | --- | --- | --- | --- | --- | --- | --- | --- | --- |
| # ALIVE | 7 | 7 | 5 | 5 | 4 | 2 | 2 | 2 | 5 | 10 | 8 | 5 | 4 |
| # DEAD | 4 | 6 | 8 | 4 | 1 | 2 | 3 | 1 | 3 | 8 | 17 | 9 | 2 |
|  |  |  |  |  |  |  |  |  |  |  |  |  |  |
|  | 2008 | 2009 | 2010 | 2011 | 2012 | 2013 | 2014 | 2015 | 2016 | 2017 | 2018 | 2019 | 2020 |
| # ALIVE | 1 | 4 | 7 | 4 | 2 | 1 | 2 | 3 | 3 | 5 | 5 | 7 | 10 |
| # DEAD | 8 | 2 | 8 | 7 | 6 | 6 | 3 | 5 | 2 | 3 | 3 | 5 | 4 |

Females entered the study the year of their first reproduction, which occurred at age one or two for nearly all individuals (Fig. A1, top-right). Because we only included reproductive females, any instance when an individual disappeared from the study site was classified as a mortality event as post-breeding dispersal is extremely rare (<1% of females, Van Vuren, unpublished). The age distribution ranged from 1 to 9 years, with the bulk of the population consisting of yearlings, followed by females aged 2, 3, or 4 years with very few instances of older females (Fig. A1, top-left). Most females produced litter sizes of 3 to 6 pups, with a fair portion of females not reproducing or failing to produce a viable litter (Fig. A1, bottom-left). The number of females in the population fluctuated widely within the study area, ranging from 5 up to 30 females a year (i.e., this number includes both reproductive and non-reproductive females; Fig. A1, bottom-right).

**Figure. A1.** Description of the age distribution, age at first reproduction distribution, litter size, and female population size for the studied population of golden-mantled ground squirrels studied at the Rocky Mountain Biological Station, in Gothic, Colorado, from 1995 to 2020.


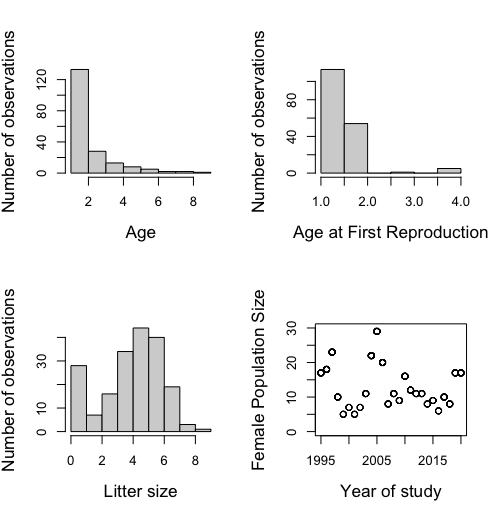

Supplement: Supplementary file 1 — Supplementary Material [file ECE3-12-e8874-s001.docx]
