# Supplementary material for: Maternal survival costs in an asocial mammal
Source: Ecol Evol. 2022 May 11;12(5):e8874. doi: 10.1002/ece3.8874 (PMC9092287; doi:10.1002/ece3.8874)
Supplement: Supplementary file 2 — Supplementary Material [file ECE3-12-e8874-s002.docx]

# **MATERNAL SURVIVAL COSTS IN AN ASOCIAL MAMMAL**

**Appendix B1.** Model selection results for Cox proportional hazard models evaluating the effect of maternal characteristics on annual survival. We considered different parameterizations of age (i), experience (defined as the number of years since an individual’s first reproductive event) (ii), residency (classified as resident if born in the study site or immigrant if immigrated in from another population) (iii), and age at first reproduction (iv).

|  | Model Set | K^1^ | Log Likelihood^2^ | AICc^3^ | Delta AICc^4^ | AICc Weight^5^ | Cumulative Weight^6^ |
| --- | --- | --- | --- | --- | --- | --- | --- |
| (i) | Age (1, 2, 3, 4+) | 1 | -361.94 | 725.90 | 0.00 | 0.51 | 0.51 |
|  | Age (Continuous) | 3 | -360.57 | 727.26 | 1.36 | 0.26 | 0.77 |
|  | Age (1, 2, 3+) | 1 | -363.07 | 728.16 | 2.26 | 0.17 | 0.94 |
|  | Age (1, 2+) | 2 | -363.05 | 730.17 | 4.27 | 0.06 | 1.00 |
| (ii) | Experience (0, 1+) | 1 | -273.95 | 549.93 | 0.00 | 0.62 | 0.62 |
|  | Experience (Continuous) | 1 | -274.46 | 550.95 | 1.02 | 0.38 | 1.00 |
| (iii) | Residency | 1 | -508.62 | -725.26 | 0.00 | 1.00 | 1.00 |
| (iv) | AFR (Continuous) | 1 | -277.10 | 556.21 | 0.00 | 0.50 | 0.50 |
|  | AFR (1, 2+)* | 1 | -277.10 | 556.21 | 0.00 | 0.50 | 1.00 |

^1^K represents the number of parameters in the model.

^2^Log Likelihood is used to predict how likely a given model is based on the available data.

^3^AICc is a score of the information lost by each model and adjusted for small sample size.

^4^Delta AICc calculates the difference in AIC score between the top model and the model being assessed.

^5^AICc Weight demonstrates the predicative power of a given model in relation to the full set of models under consideration.

^6^Cumulative Weight shows the additive effect of the AICc weights as more model AICc values are considered.

* Note that “Age (Continuous)” and “Age (1, 2+) reach the same results because AFR only includes 1 or 2-year-olds.

**Appendix B2.** Model selection results for Cox proportional hazard models testing for the effect of social context factors on annual survival. We considered different parameterizations of litter size (i), litter sex ratio (ii), collective litter mass (iii), day of litter emergence (iv), local density of breeding females (v), local density of related breeding female (vi), local density of unrelated breeding females (vii), and local population density of total breeding and non-breeding females.

|  | Model Set | K^1^ | Log Likelihood^2^ | AICc^3^ | Delta AICc^4^ | AICc Weight^5^ | Cumulative Weight^6^ |
| --- | --- | --- | --- | --- | --- | --- | --- |
| (i) | Size (Continuous) | 1 | -315.64 | 633.30 | 0.00 | 0.38 | 0.38 |
|  | Size (0, 1+) | 1 | -315.67 | 633.36 | 0.06 | 0.37 | 0.75 |
|  | Size (0, 1-2, 3-4, 5+) | 3 | -314.31 | 634.75 | 1.44 | 0.19 | 0.94 |
|  | Size (0, 1-3, 4-5, 6+) | 3 | -315.41 | 636.96 | 3.65 | 0.06 | 1.00 |
| (ii) | Ratio (Continuous) | 1 | -243.55 | 489.12 | 0.00 | 0.86 | 0.86 |
|  | Ratio (Categorical) | 3 | -243.33 | 492.81 | 3.69 | 0.14 | 1.00 |
| (iii) | Mass (Continuous) | 1 | -82.02 | 166.10 | 0.00 | 0.66 | 0.66 |
|  | Mass (Lite, Moderate, Heavy) | 2 | -81.64 | 167.45 | 1.34 | 0.34 | 1.00 |
| (iv) | Emergence (Continuous) | 1 | -263.15 | 528.33 | 0.00 | 0.97 | 0.97 |
|  | Emergence (Early, Moderate, Late) | 3 | -264.67 | 535.48 | 7.15 | 0.03 | 1.00 |
| (v) | Breeders (0-1, 2+) | 1 | -485.89 | 973.79 | 0.00 | 0.54 | 0.54 |
|  | Breeders (Continuous) | 1 | -486.39 | 974.80 | 1.01 | 0.33 | 0.87 |
|  | Breeders (0, 1-2, 3+) | 2 | -486.31 | 976.67 | 2.87 | 0.13 | 1.00 |
| (vi) | Kin (Continuous) | 1 | -480.59 | 963.19 | 0.00 | 0.50 | 0.50 |
|  | Kin (0-1, 2+) | 1 | -481.14 | 964.30 | 1.11 | 0.29 | 0.78 |
|  | Kin (0, 1-2, 3+) | 2 | -480.40 | 964.86 | 1.67 | 0.22 | 1.00 |
| (vii) | Non-Kin (Continuous) | 1 | -481.89 | 965.79 | 0.00 | 0.43 | 0.43 |
|  | Non-Kin (0-1, 2+) | 1 | -481.90 | 965.82 | 0.04 | 0.42 | 0.84 |
|  | Non-Kin (0, 1-2, 3+) | 2 | -481.86 | 967.78 | 1.99 | 0.16 | 1.00 |
| (viii) | Population (Continuous) | 1 | -485.28 | 972.58 | 0.00 | 0.50 | 0.50 |
|  | Population (0-1, 2+) | 1 | -485.75 | 973.52 | 0.94 | 0.31 | 0.81 |
|  | Population (0, 1-2, 3+) | 2 | -485.22 | 974.49 | 1.91 | 0.19 | 1.00 |

^1^K represents the number of parameters in the model.

^2^Log Likelihood is used to predict how likely a given model is based on the available data.

^3^AICc is a score of the information presented by the model and adjusted for small sample size.

^4^Delta AICc calculates the difference in AIC score between the top model and the model being assessed.

^5^AICc Weight demonstrates the predicative power of a given model in relation to the full set of models under consideration.

^6^Cumulative Weight shows the additive effect of the AICc weights as more model AICc values are considered.

**Appendix B3.** Model selection results for Cox proportional hazard models testing for the effect of environmental context factors on annual survival. We considered different parameterizations of first day of bare ground (i), first day of permanent snow cover (ii), length of the growing season (iii), amount of winter snowfall (iv), amount of summer rainfall during June and July (v), average summer temperature during June and July (vi), and number of days above 25^o^C during June and July (vii).

|  | Model Set | K^1^ | Log Likelihood^2^ | AICc^3^ | Delta AICc^4^ | AICc Weight^5^ | Cumulative Weight^6^ |
| --- | --- | --- | --- | --- | --- | --- | --- |
| (i) | Bare (Early, Moderate, Late) | 2 | -521.41 | 1046.86 | 0.00 | 0.71 | 0.71 |
|  | Bare (Continuous) | 1 | -523.31 | 1048.64 | 1.78 | 0.29 | 1.00 |
| (ii) | Covered (Continuous) | 1 | -521.35 | 1044.72 | 0.00 | 0.60 | 0.60 |
|  | Covered (Early, Moderate, Late) | 2 | -520.74 | 1045.53 | 0.80 | 0.40 | 1.00 |
| (iii) | Length (Short, Medium, Long) | 2 | -520.84 | 1045.74 | 0.00 | 0.55 | 0.55 |
|  | Length (Continuous) | 1 | -522.07 | 1046.16 | 0.43 | 0.45 | 1.00 |
| (iv) | Snow (Low, Moderate, High) | 1 | -522.94 | 1047.89 | 0.00 | 0.66 | 0.66 |
|  | Snow (Continuous) | 2 | -522.60 | 1049.25 | 1.36 | 0.34 | 1.00 |
| (v) | Rain (Continuous) | 1 | 949.74 | 0.00 | 0.50 | 0.50 | -473.86 |
|  | Rain (Low, Moderate, High) | 2 | 949.74 | 0.00 | 0.50 | 1.00 | -472.85 |
| (vi) | Temperature (Continuous) | 1 | -522.43 | 1046.88 | 0.00 | 0.78 | 0.78 |
|  | Temperature (Low, Moderate, High) | 2 | -522.67 | 1049.40 | 2.51 | 0.22 | 1.00 |
| (vii) | 25^o^C Days (Continuous) | 1 | -522.84 | 1047.70 | 0.00 | 0.79 | 0.79 |
|  | 25^o^C Days (Low, Moderate, High) | 2 | -523.14 | 1050.33 | 2.64 | 0.21 | 1.00 |

^1^K represents the number of parameters in the model.

^2^Log Likelihood is used to predict how likely a given model is based on the available data.

^3^AICc is a score of the information presented by the model and adjusted for small sample size.

^4^Delta AICc calculates the difference in AIC score between the top model and the model being assessed.

^5^AICc Weight demonstrates the predicative power of a given model in relation to the full set of models under consideration.

^6^Cumulative Weight shows the additive effect of the AICc weights as more model AICc values are considered.
