# Supplementary material for: Maternal survival costs in an asocial mammal
Source: Ecol Evol. 2022 May 11;12(5):e8874. doi: 10.1002/ece3.8874 (PMC9092287; doi:10.1002/ece3.8874)
Supplement: Supplementary file 3 — Supplementary Material [file ECE3-12-e8874-s003.docx]

# **MATERNAL SURVIVAL COSTS IN AN ASOCIAL MAMMAL**

**Appendix C. Impact of maternal emergence body mass in year t on the maternal mortality hazard from year to to t+1.**

Body mass at emergence was collected for about 60% of the ‘female-year’ observations in our dataset. This was too much missing data to consider including body mass as a covariate in our existing model set. We built a separate model to explore the effect of emergence body mass on annual survival based on the subset of data we had. We observed that neither a continuous and standardized (**Table C1**), nor a categorical effect of spring emergence body mass (i.e. below versus above average; **Table C2**) had a significant impact on the maternal mortality hazard.

**Table C1.** Effect of body mass at emergence (continuous and standardized) on the maternal hazard rate based on 155 observations and 89 mortality events:

|  | coef | exp(coef) | se(coef) | z | Pr(>\|z\|) |
| --- | --- | --- | --- | --- | --- |
| Mass et Emergence | -0.1033 | 0.9019 | 0.1138 | -0.907 | **0.364** |
|  |  |  |  |  |  |
|  | exp(coef) | exp(-coef) | lower .95 | upper .95 |  |
| Mass et Emergence | 0.9019 | 1.109 | 0.7216 | 1.127 |  |

**Table C2.** Effect of body mass at emergence (categorical: below versus above average, where above is set at the reference hazard where exp(coef)=1) on the maternal hazard rate based on 155 observations and 89 mortality events:

|  | coef | exp(coef) | se(coef) | z | Pr(>\|z\|) |
| --- | --- | --- | --- | --- | --- |
| Mass et Emergence (below average) | 0.2298 | 1.2584 | 0.2237 | 1.027 | 0.304 |
|  |  |  |  |  |  |
|  | exp(coef) | exp(-coef) | lower .95 | upper .95 |  |
| Mass et Emergence (below average) | 1.258 | 0.7947 | 0.8117 | 1.951 |  |
